# The prediction of haemophilic arthropathy progression based on MRI findings and clinical characteristics

**DOI:** 10.1186/s13023-025-03716-1

**Published:** 2025-04-18

**Authors:** Lu Zhang, Jinxia Guo, Shufang Wei, Jiajia Li, Yincong Dou, Tianming Cheng, Yinghui Ge, Tuo Zhang

**Affiliations:** 1 Department of Medical Imaging, Henan Provincial People’s Hospital, People’s Hospital of Zhengzhou University, Zhengzhou, Henan China; 2 GE Healthcare MR Research, Beijing, China; 3 Department of Radiology, Fuwai Central China Cardiovascular Hospital, Zhengzhou, Henan China

**Keywords:** Haemophilic arthropathy, Magnetic resonance imaging, Progression, Prediction, Synovial hypertrophy

## Abstract

**Objective:**

To identify magnetic resonance imaging (MRI) and clinical characteristics that are closely associated with the progression of haemophilic arthropathy (HA) after different therapies and to establish a prediction model for HA progression using Cox proportional hazards regression, thus facilitating the development of personalized clinical replacement therapy plans.

**Materials and methods:**

Retrospective clinical and imaging data were collected from HA patients registered at the Henan Provincial Registration Management Center of Haemophilia from December 2010 to May 2023. The inclusion criteria were joints with a history of haemorrhage and initial/posttreatment reevaluation with X-ray and MRI. Joints with severe damage (i.e., a Pettersson score > 6) were excluded. Joint disease progression was defined as a > 1-point increase in the Pettersson score. Progression-free survival (PFS) was the primary outcome. MRI observations revealed joint effusion, synovial hypertrophy, haemosiderin deposition, bone destruction or cystic degeneration at the joint margins, and cartilage destruction. Age, body mass index (BMI), factor VIII (FVIII) activity, activated partial thromboplastin time (APTT), prothrombin time (PT), therapy type, annual joint bleeding rate (AJBR), and the Haemophilia Joint Health Score (HJHS) were also assessed. Subsequently, univariate and multivariate Cox proportional hazards regression models were employed to analyse the clinical and imaging characteristics influencing HA progression. Factors with a *P* < 0.15 in univariate analysis were subsequently included in the multivariate analysis. The impact of various imaging and clinical characteristics on PFS was assessed via Kaplan‒Meier (K-M) survival curves.

**Results:**

This study included 98 joints across 65 patients. During the follow-up period, 63 joints exhibited progression. Both univariate and multivariate Cox analyses revealed that MRI-detected synovial hypertrophy (MRI-SP) was an independent risk factor for HA progression. Incorporating BMI into the model improved its predictive performance (Model 1: c-index = 0.671, *P* < 0.01). Spearman’s correlation analysis revealed strong correlations between baseline MRI-SP and detected haemosiderin deposition (*r* = 0.73) as well as AJBRs (*r* = 0.66). K-M survival curves indicated that patients receiving prophylactic treatment and those with less severe MRI-SP had better progression-free survival.

**Conclusion:**

MRI-detected synovial hypertrophy is an independent risk factor for HA progression. The predictive model, which includes BMI as a covariate for assessing the risk of HA progression, can serve as an auxiliary tool for developing personalized treatment plans for HA patients.

## Introduction

Haemophilia is a lifelong condition characterized by spontaneous or minor trauma-induced bleeding due to a deficiency in clotting factors, with joint haemorrhage being the most common manifestation. Repeated joint bleeding leads to pain, deformities, and functional impairments, ultimately leading to haemophilic arthropathy (HA) [1]. Inadequate treatment can increase the incidence of joint diseases in moderate to severe cases by 45% among patients between the ages of 3 and 6, resulting in school-age children dropping out or being unable to participate in activities [2].

Currently, there is no cure for HA, and it requires lifelong treatment. Factor replacement therapy is the only effective measure, encompassing both on-demand and prophylactic treatments. On-demand treatment, which is administered after an acute bleeding episode, aims to stop the bleeding. However, by this stage, intra-articular haemorrhage has already triggered inflammatory processes in the synovium and cartilage, leading to irreversible joint damage [3]. While low-dose prophylactic treatment is cost-effective, it may not effectively prevent bleeding in some patients [4]. Early identification of risk factors for HA progression during regular joint assessments can significantly reduce the occurrence of bleeding events and delay HA progression by adjusting the timing or dosage of prophylactic treatment.

MRI is recognized as the gold standard for the assessment of HA joints [5]. Recent studies have explored MR imaging techniques to evaluate joint structure and the efficacy of gene transfer treatment methods [6–8]. Additionally, studies have analysed correlations between bleeding phenotypes, physical examinations, MRI findings and joint function scores [9]. However, most of these studies are cross-sectional, and few longitudinal imaging studies tracking HA joints exist to evaluate the potential of MRI in predicting HA progression. This study retrospectively analysed MRI and clinical data from target joints of patients over more than 10 years of age to identify MRI and clinical risk factors closely associated with HA progression in patients who underwent different therapies. Furthermore, we aimed to explore a prediction model for HA progression using Cox proportional hazards regression, which may be valuable for personalized clinical replacement therapy plans.

## Materials and methods

### Study population and Follow-up methods

We conducted a retrospective analysis of clinical and imaging data for haemophilia patients registered at the Henan Provincial Registration Management Center of Haemophilia from December 2010 to May 2023. This study received approval from our institution’s ethics committee (Approval No. 2017-No. 49). The inclusion criteria were as follows: (1) patients with haemophilia who regularly attended follow-up at the Haemophilia Center; (2) patients whose joints, including the knees, ankles, elbows, or hips, had a history of bleeding; and (3) patients who underwent initial and posttreatment evaluations with X-ray and MRI to assess joint conditions.

**Fig. 1:**
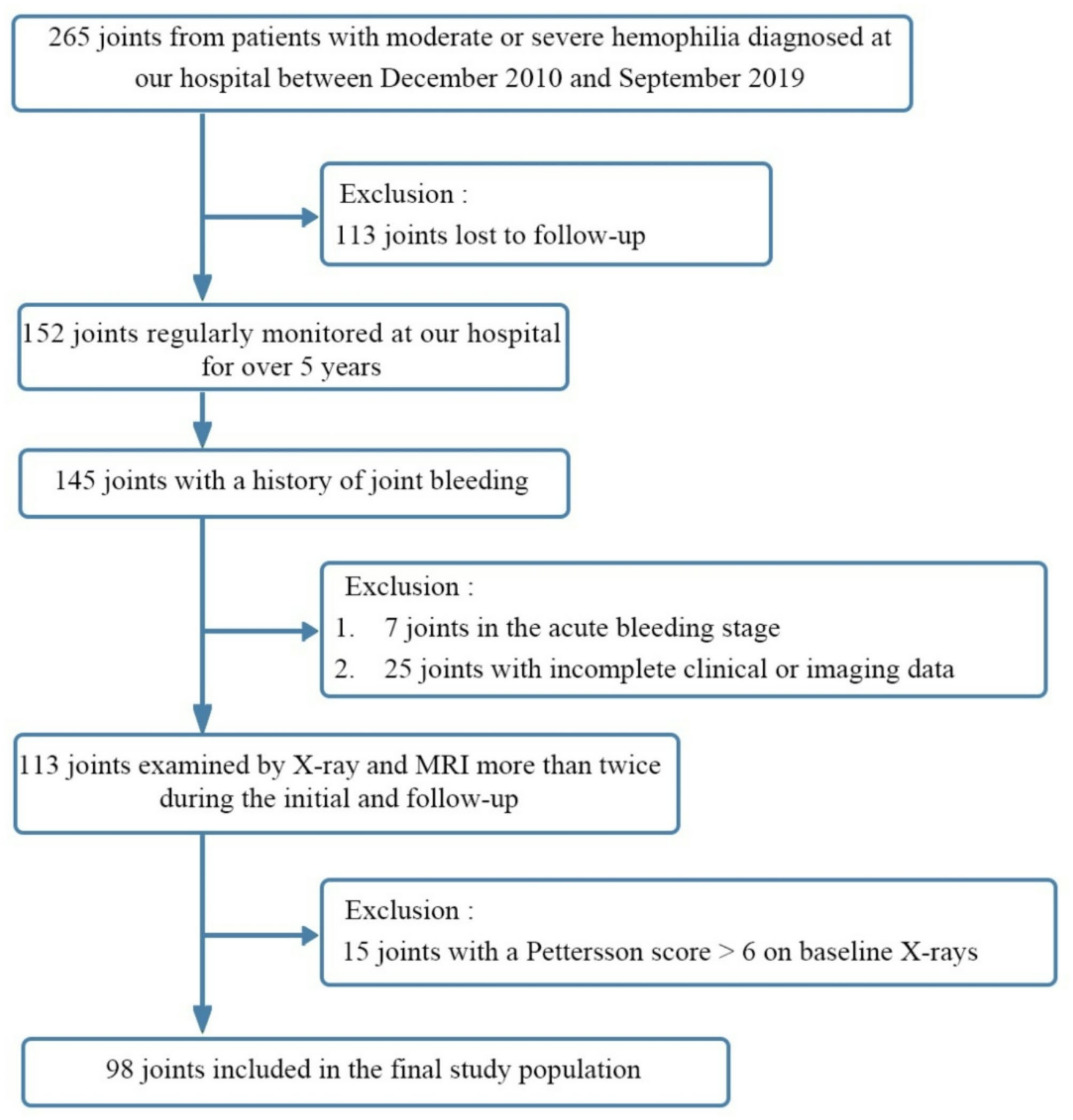
Flowchart of Study Population Screening

The exclusion criteria were as follows: (1) joints with severe HA damage, indicated by an X-ray Pettersson score > 6 [10], as these joints are no longer suitable for clinical substitution treatment); (2) joint diseases resulting from other conditions, such as fractures, tumours, osteoarthritis, or rheumatoid arthritis; (3) patients who had undergone orthopaedic surgery; and (4) patients who did not regularly attend follow-up appointments, leading to incomplete clinical or imaging data. Ultimately, 64 patients with 98 target joints were included in the analysis. A detailed flowchart of the study cohort is presented in Fig. 1.

During follow-up, an increase of one or more points in the X-ray Pettersson score was defined as joint progression. The time interval between the first X-ray examination and the point at which the Pettersson score increased was recorded for progression-free survival (PFS) analysis. For joints without progression, the interval was defined as the duration from the first to the most recent X-ray examination.

### Clinical data collection

A radiologist who was blinded to the clinical outcomes and imaging scores extracted baseline clinical information from patient records, including age, BMI, factor VIII (FVIII) activity, activated partial thromboplastin time (APTT), prothrombin time (PT), therapy type, annual joint bleeding rate (AJBR), and the Haemophilia Joint Health Score (HJHS). The HJHS assessment was based on version 2.1 [11] and evaluated joint function across eight dimensions and four types of gait abilities.

### Imaging data

#### Imaging acquisition

Routine anteroposterior and lateral radiographs of the joints were obtained using the GE Discovery XR650 digital radiography system. MRI was performed with the GE 3.0T Discover 750 MRI system, which uses specific coils for each joint: a knee coil for knee scans, an ankle coil for ankle scans, and a flexible surface coil for elbow scans.

For the knee joint, the T1-weighted imaging (T1WI) parameters were TR = 310 ms and TE = 11 ms, whereas the proton density-weighted imaging with fat saturation (PDWI-FS) parameters were TR = 4200 ms and TE = 36 ms. For the ankle joint, the T1WI parameters were TR = 416 ms and TE = 11 ms, and the PDWI-FS parameters were TR = 4180 ms and TE = 33 ms. For the elbow joint, the T1WI parameters were TR = 498 ms and TE = 10 ms, whereas the PDWI-FS parameters were TR = 3000 ms and TE = 22 ms. The field of view (FOV) was set to 16 cm × 16 cm, with a slice thickness of 3.5 mm and an interslice gap of 0.4 mm.

#### Imaging observation indicators

All baseline and follow-up X-ray and MR images were analysed using the Picture Archiving and Communication System (PACS). Two experienced musculoskeletal radiologists (Assessor 1 and Assessor 2, with 8 and 10 years of experience, respectively) independently evaluated the X-ray and MRI scans, employing the Pettersson scoring system and the International Prophylaxis Study Group (IPSG) [12] score sheet as standards (Table 1). They were blinded to the clinical data. Any increase in the X-ray Pettersson score was considered to indicate joint disease progression.

**Table 1:** The additive IPSG MRI scale for haemophilic arthropathy

Imaging Characteristic	Severity Grading	Score
Effusion/haemarthrosis	Mild	1
	Moderate	2
	Severe	3
Synovial hypertrophy	Mild	1
	Moderate	2
	Severe	3
Haemosiderin	Mild	1
	Moderate	2
	Severe	3
Surface erosions involving subchondral cortex or joint margins	Presence of surface erosion	1
	Half or more of the articular surface eroded in at least one bone	2
Subchondral cysts	At least one subchondral cyst	1
	Subchondral cysts in at least two bones, or cystic changes involving a third or more of the articular surface in at least one bone	2
Cartilage degeneration	Reduction in joint cartilage thickness	1
	Loss of half or more of the total volume of joint cartilage in at least one bone	2
	Full-thickness loss involving joint cartilage in at least some area in at least one bone	3
	Full-thickness loss of joint cartilage including at least one half of the joint surface in at least one bone	4
Total		17

IPSG: International Prophylaxis Study Group

### Statistical analysis

The interclass correlation coefficient (ICCs) was calculated to assess the consistency of the MRI IPSG score, X-ray Pettersson score and clinical joint function HJHS score among observers. The median (range) or percentage of clinical features was then computed. Baseline clinical and MRI characteristics, including age, BMI, clotting factor concentration, prothrombin time (PT), activated partial thromboplastin time (APTT), therapy type, annual joint bleeding rates (AJBRs), and imaging features, were compared between the progression and nonprogression groups. The Mann‒Whitney test was applied to numerical variables due to nonnormality, whereas the chi‒square test was used for categorical variables. Univariate and multivariate Cox proportional hazard regression analyses were conducted to evaluate the predictive potential of MRI features and clinical risk factors for HA progression prediction. Variables with a *P* < 0.15 in the univariate analysis were subsequently included in the stepwise multivariate analysis to develop the prediction model. Spearman’s correlation analysis was conducted between pairs of clinical and imaging indicators. Survival curves were generated using the Kaplan‒Meier (K-M) method and compared using the log-rank test. For numerical variables, the optimal threshold was determined using the maximal selected log-rank statistic (i.e., the lowest *P* value) to generate K-M curves [13]. A two-tailed *P* value < 0.05 was considered to indicate statistical significance. Statistical analyses were performed using SPSS software version 21.0 and the R package (version 3.6.0), which utilize the “survival” and “rms” packages.

## Results

### Interrater reliability

The interrater reliability, represented by the ICC coefficient, between the two radiologists for HA joint imaging features and joint function scores is shown in Table 2. High consistency was observed across all the characteristics, and only the results from the first radiologist were utilized in subsequent analyses.

**Table 2:** Assessor consistency analysis of haemophilic arthropathy X-ray and MRI scoring

Parameters	Observer 1 Score (percentage)	Observer 2 Score (percentage)	ICC (95% CI)
*(MRI features)*			
Effusion/haemarthrosis	0 (5/98, 5.1%)	0 (5/98, 5.1%)	0.9954(0.9932–0.9971)
	1 (59/98, 60.2%)	1 (59/98, 60.2%)	
	2 (23/98, 23.5%)	2 (24/98, 24.5)	
	3 (11/98, 11.2%)	3 (10/98, 10.2%)	
Synovial hypertrophy	0(14/98, 14.3%)	0 (14/98, 14.3%)	0.9927(0.9891–0.9951)
	1(38/98, 38.8%)	1 (39/98, 29.6%)	
	2(38/98, 38.8%)	2 (37/98, 37.8%)	
	3(8/98, 8.2%)	3 (8/98, 8.2%)	
Haemosiderosis	0(28/98, 28.6%)	0 (28/98, 28.6%)	0.9862(0.9794–0.9908)
	1(30/98, 30.6%)	1 (31/98, 31.6%)	
	2(23/98, 23.5%)	2 (24/98, 24.5%)	
	3(17/98, 17.3%)	3 (15/98, 15.3%)	
Bone erosion	0(40/98, 40.8%)	0 (40/98, 40.8%)	0.9928(0.9892–0.9951)
	1(29/98, 29.6%)	1 (28/98, 29.6%)	
	2(29/98, 29.6%)	2 (30/98, 30.6%)	
Subarticular bone cysts	0(49/98, 50.0%)	0 (49/98, 50.0%)	1
	1(19/98, 19.4%)	1 (19/98, 19.4%)	
	2(30/98, 30.6%)	2 (30/98, 30.6%)	
Cartilage degradation	0 (30/98, 30.6%)	0 (30/98, 30.6%)	1
	1 (15/98, 15.3%)	1 (15/98, 15.3%)	
	2 (23/98, 23.5%)	2 (23/98, 23.5%)	
	3 (14/98, 14.3%)	3 (14/98, 14.3%)	
	4(16/98, 16.3%)	4 (16/98, 16.3%)	
MRI IPSG score*	8(0–16)	8(0–15)	0.9979(0.9969–0.9986)
X-ray Pettersson score*	2(0–6)	2(0–6)	0.997(0.9968–0.9985)
HJHS score*	6(0–18)	6(0–18)	1

Note: * Median (range)

### Baseline clinical and imaging characteristics

Sixty-five male haemophilia patients with a total of 98 joints (72 knee joints, 18 ankle joints, 4 elbow joints, and 4 hip joints) were included. The median age was 12 years (range 3–46), and the median BMI was 20.71 (range 14.87–28.81). Among the patients, 36 had severe haemophilia (FVIII activity < 1%), and 29 had moderate haemophilia (median FVIII activity 4%, range 2-5%). According to the Chinese expert consensus on the diagnosis and treatment of haemophilia [14], 17 patients received low-dose prophylactic treatment (11 IU/kg per dose; IQR, 10–13 IU/kg, twice a week) starting at a median age of 4 years (range 0.9–5.5 years). The remaining 48 patients received on-demand treatment, which involved administering 1 IU/kg body weight of FVIII to achieve an in-body FVIII activity of 10%~20%, given every 12 h (Table 3).

**Fig. 2:**
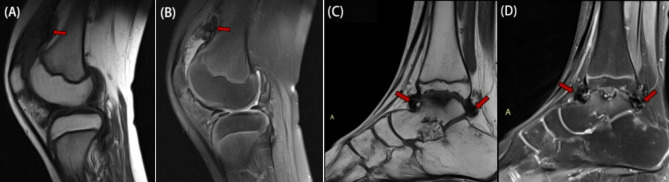
MRI Findings in Two Cases of Haemophilic Arthropathy. Figures 3A-B: An 11-year-old male with a history of haemophilia A since childhood, presenting with recurrent bleeding, swelling, and pain in the left knee. Sagittal T1-weighted imaging (T1WI) (**A**) and proton density-weighted imaging with fat saturation (PD-FS) (**B**) of the left knee joint. Figures 3C-D: A 10-year-old male with a similar history of haemophilia A since childhood, presenting with recurrent bleeding, swelling, and pain in the right ankle. Sagittal T1WI (**C**) and PD-FS (**D**) images of the right ankle joint are displayed. Haemosiderin appears as areas of extremely low signal intensity on both T1WI and PD-FS images. Haemosiderin deposition is closely associated with synovial hypertrophy on MRI, primarily located in the joint capsule folds and hypertrophic synovial tissue (red arrows)

All study joints had a history of bleeding. The most common MRI findings included joint effusion in 93 (95%) joints, synovial hypertrophy in 84 (86%) joints, and haemosiderin deposition in 70 (71%) joints. Synovial hypertrophy and haemosiderin deposition were frequently observed together (Fig. 2): among the 84 joints with synovial hypertrophy, 69 (82.1%) also presented haemosiderin deposition, and among the 70 joints with haemosiderin deposition, 69 (98.6%) presented synovial hypertrophy.

**Table 3:** Baseline patient and joint features

Patient features	Median (Range) or *n* (%)				
N	65				
Age, years	12(3–46)				
BMI	20.71(14.87–28.81)				
Grade	36(55%) severe haemophilia	29(45%) moderate haemophilia			
Therapy	17(26%) On prophylaxis	48 (74%) On demand			
**Joint features**					
N	98(72 knees, 18 ankles, 4 elbows, 4 hips)				
HJHS score	6(0–18)				
Pettersson score	2(0–6)				
*(MRI score)*	0	1	2	3	4
Joint effusion	5 (2%)	59(60%)	23(23%)	11(11%)	
Synovial hypertrophy	14(14%)	38(39%)	38(39%)	8(8%)	
Hemosiderosis	28(29%)	30(31%)	23(23%)	17(17%)	
Bone erosion	40(41%)	29(30%)	29(30%)		
Subarticular bone cyst	49(50%)	19(19%)	30(31%)		
Cartilage degradation	30(31%)	15(15%)	23(23%)	14(14%)	16(16%)

### Follow-Up and clinical outcomes

**Fig. 3:**
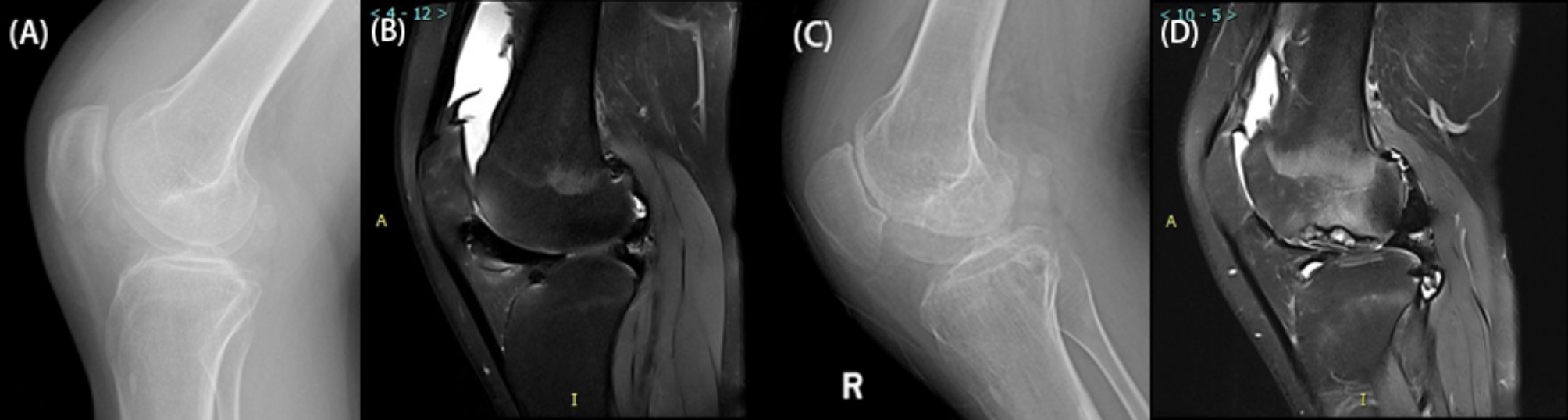
Haemophilic knee joint of a 20-year-old male with a history of haemophilia A since childhood in the progression group. He experienced recurrent bleeding and pain in the right knee joint and received factor replacement therapy on demand. Figures A-B show the initial X-ray (Pettersson score = 2) and MRI (synovial hypertrophy score = 2, indicating a high risk of progression) results on December 14, 2017. Figures C-D depict the X-ray (Pettersson score = 4) and MR images taken on May 25, 2020. An increase in the Pettersson score indicates joint disease progression, whereas MRI reveals increased destruction of joint cartilage and subchondral bone

Among the 98 patients, 63 (64.3%) experienced progression of joint disease during follow-up, with a median PFS of 554 days (range 110–1884 days). Among the 63 progressed joints, the median increase in the Pettersson score was 2 points (range 1–8 points) (Fig. 3A-D).

#### Cox univariate analysis related to HA joint progression

Univariate analysis indicated that MRI-derived joint effusion/hemarthrosis, synovial hypertrophy, and haemosiderin deposition were significant prognostic risk factors (*P* < 0.05). In contrast, the clinical risk factors, including PT, APTT, AJBR, and HJHS scores, were not significant (*P* > 0.05) (Table 4).

**Table 4:** Univariate Cox regression analyses of progression-free survival in patients with haemophilic arthropathy

Variables	HR	95% CI	*P* value	C-index
Age(years)	0.954	(0.916–0.994)	**0.0228**	0.534
BMI	0.826	(0.695–0.9811)	**0.030**	0.645
Grade	1.164	(0.5326–2.549)	0.704	0.492
PT	0.973	(0.655–1.446)	0.892	0.551
APTT	1.004	(0.990–1.018)	0.584	0.512
AJBRs	1.020	(0.992–1.049)	0.159	0.556
*(MRI features)*				
Joint effusion	1.402	(1.054–1.865)	**0.020**	0.577
Synovial hypertrophy	1.470	(1.036–2.085)	**0.031**	0.578
Hemosiderosis	1.290	(0.995–1.672)	0.055	0.575
Bone erosion	0.962	(0.667–1.387)	0.835	0.527
Subarticular bone cyst	1.009	(0.749–1.359)	0.949	0.491
Cartilage degradation	1.054	(0.878–1.266)	0.572	0.526
MRI IPSG score	1.043	(0.989–1.101)	0.123	0.554
X-ray Pettersson score	0.934	(0.813–1.072)	0.330	0.557
HJHS score	1.006	(0.962–1.053)	0.789	0.482

Note: AJBRs: annualized joint bleeding rates; PT: prothrombin time; APTT: activated partial thromboplastin time

#### Cox multivariate analysis results related to HA progression

The multivariate analysis (Table 5) revealed that synovial hypertrophy (MRI-synovial hypertrophy, MRI-SP) was an independent risk factor for HA progression. When BMI was included as a covariate for MRI-SP, the concordance index (c-index) improved to 0.671 (*P* < 0.01) compared with that of MRI-SP alone (0.578). The model combining BMI and the IPSG score was also evaluated, yielding a c-index of 0.668.

**Table 5:** Multivariate Cox regression for progression-free survival prediction

Models	HR(95% CI)	Z	*P* value	C -index
Model 1: BMI + MRI synovial hypertrophy				
BMI	0.822(0.690–0.979)	-2.202	0.028	0.671
*MRI synovial hypertrophy*	1.433(1.105–1.857)	2.716	0.007	
Model 2: BMI + MRI IPSG score				
BMI	0.791(0.664–0.942)	-2.624	0.009	0.668
*MRI IPSG score*	1.081(1.022–1.144)	2.738	0.006	

#### Spearman’s correlation analysis

Spearman’s correlation analysis revealed that the correlation coefficients for MRI-SP with haemosiderin deposition, AJBRs and HJHS were 0.73 (*P* < 0.05), 0.66 (*P* < 0.05) and 0.57 (*P* < 0.05), respectively. HJHS was found to be significantly correlated with AJBRs (coefficient = 0.60, *P* < 0.05) as was the Pettersson score (coefficient = 0.69, *P* < 0.05).

#### HA Kaplan‒Meier (K-M) curve

**Fig. 4:**
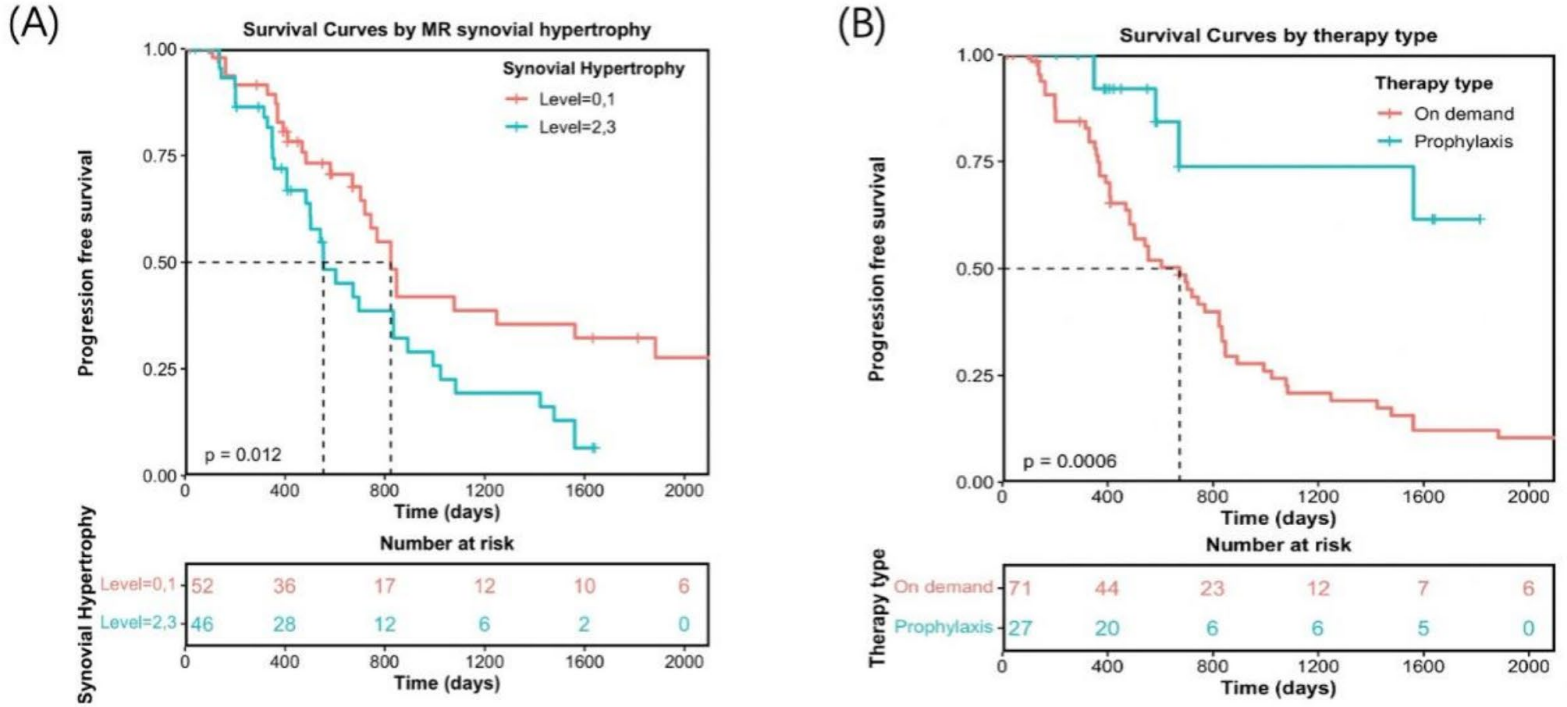
Kaplan-Meier curves for annual progression-free survival (PFS) based on MRI synovial hypertrophy (**A**) and type of treatment (**B**)

The K-M curves (Fig. 4A) indicated that patients with an MRI-SP score of 0–1 exhibited better progression-free survival than those with a score of 2–3, with a statistically significant difference (*P* < 0.05). Figure 4B shows that patients receiving “prophylactic treatment” had a significantly longer progression-free survival period than did those receiving “on-demand treatment” (*P* < 0.05).

## Discussion

We conducted a retrospective analysis of the clinical and imaging features of haemophilia patients admitted to the haemophilia Registration Management Center over more than 10 years to identify risk factors associated with HA joint progression. Our study revealed that MRI-SP is an independent risk factor influencing HA progression and that predictive accuracy improves when BMI is included as a covariate for MRI-SP.

Cox regression analysis indicated that patients with more severe synovial hypertrophy faced a greater risk of joint bleeding, which accelerated arthropathy progression. Kaplan‒Meier curves (Fig. 4 (A)) demonstrated that patients with MRI-SP scores of 0–1 had significantly better PFS than those with scores of 2–3 (*P* = 0.012). Research indicates that more than 90% of patients on prophylaxis experience at least one joint with chronic changes before the age of 40 [15, 16]. Following acute joint bleeding, approximately one week is needed for normal synovial lining cells to clear blood from the joint [7, 17]. However, in haemophilia patients, factors such as abnormal remodelling of the endothelial basement membrane, inhibition of coagulation cascade initiation, and a hyperactive fibrinolytic system can prolong and even cause repeated bleeding events, exceeding the clearance capacity of synovial lining cells [18].

Synovitis is mediated by a series of cytokine-mediated inflammatory responses. Research by Sen [19] et al. revealed that joint bleeding activates various inflammatory cytokines, triggering the NF-κB pathway. The activated NF-κB transcription factor exacerbates synovitis through the proinflammatory cytokines IL-1β and TNFα. VEGF molecules produced within the joint cavity after bleeding further promote synovial angiogenesis [20], creating a vicious cycle of “bleeding-synovitis-rebleeding”. Spearman’s correlation analysis revealed strong associations between synovial hypertrophy, haemosiderin deposition, and joint bleeding on MRI. The iron produced by macrophages and synovial cells after phagocytosis of damaged red blood cells presented as haemosiderin, ferritin, and free iron, thus contributing to and exacerbating the pathogenesis of HA by inducing the abnormal expression of genes regulating cell proliferation and apoptosis [21]. Therefore, it may be necessary to implement more aggressive prophylactic treatment plans to minimize the risk of rebleeding and delay HA progression for patients with severe MRI-detected synovial hypertrophy (scores 2–3). The number of joint bleeding events at the time of MRI scanning was recorded from patient files; only bleeds that were registered and treated were counted. Our research indicated that AJBRs were not identified as independent risk factors for HA progression, possibly because of subclinical bleeding events that were not recorded by patients or clinicians.

In our combined prediction model, BMI was considered a covariate, significantly improving the model performance to 0.671 (*P* < 0.01) compared with MRI-SP alone, which had a c-index of 0.578. A study conducted in the United States indicated that obesity negatively impacts treatment costs and overall management of haemophilia, including health care expenses and chronic complications [22]. In our study, the hazard ratio (HR) for BMI was less than 1, indicating that BMI is a protective factor that significantly influences HA progression. This finding may be attributed to the fact that the patients in this study primarily received on-demand treatment, resulting in a much higher frequency of joint bleeding than in U.S. patients who received prophylactic treatment. Additionally, our cohort generally had low BMI values, with very few obese patients. Haemophilia is a chronic consumptive disease, and patients with long-term chronic HA in this study were often in a cachectic state. Consequently, those with excessively low BMIs typically receive less effective replacement therapy, face a greater risk of bleeding, and experience increased HA progression. In contrast, patients with a relatively higher BMI represented a more stable disease phase, which was associated with a lower risk of progression. Previous research has shown that overweight or obese RA patients have a significantly lower risk of rapid radiographic progression (RRP) within 5 years than normal weight patients do [23]. These findings are consistent with the results of the current study, which suggest that BMI serves as a protective factor against HA progression. Furthermore, our analysis revealed that Model 2 (BMI + “MRI-IPSG score”) had a c-index of 0.668 (*P* < 0.01), comparable to the predictive efficacy of Model 1 based on MRI-SP, thus highlighting the IPSG score as an important clinical assessment indicator. The IPSG scoring system, designed to assess joint condition in haemophilia patients, provides a comprehensive overview of joint bone and soft tissue [24], serving as an intuitive indicator for assessing the high risk of HA progression.

Survival analysis of HA patients via K-M curves revealed that patients receiving “prophylactic treatment” experienced significantly prolonged progression-free survival. This finding underscores the importance of treatment methods as crucial factors influencing HA progression and prognosis. High-dose prophylactic treatment is common in most developed countries; however, in China, the current treatment landscape primarily consists of on-demand therapy, with only a small portion of patients receiving low-dose prophylaxis [25]. Compared with developed nations, China has a higher incidence and overall severity of HA [26]. In our study, 48 patients (73.9%) received on-demand treatment, whereas 17 patients (26.1%) received low-dose prophylaxis. The full implementation of standard-dose prophylaxis remains challenging, thus highlighting the need for our research on the early detection of risk factors for HA progression as well as the development of a prediction model based primarily on on-demand treatment and a limited number of low-dose prophylaxis cases. By optimizing the current substitution treatment plans in a cost-effective manner, this study aims to help reduce the risk of joint bleeding and delay the progression of HA.

## Limitations

First, this study is a provincial single-centre retrospective analysis, resulting in a relatively small sample size. Large-scale, multicentre studies are needed for further validation of the model. Additionally, the majority of patients in this study received on-demand treatment, with only a small portion receiving low-dose prophylaxis. Future studies should aim to expand the study population and develop predictive models for groups with different treatment protocols.

## Conclusion

MRI-SP is an independent risk factor for the progression of HA in joints. The MRI-SP predictive model, which incorporates BMI as a covariate, may be useful for estimating the severity and likelihood of HA progression. The model could aid in planning personalized factor replacement therapy, help reduce the occurrence of bleeding events and delay the progression of HA.

## Data Availability

The datasets generated and/or analysed during this study are not publicly available due to patient privacy concerns, but they can be obtained from the corresponding author upon reasonable request.

## Abbreviations

HA: Haemophilic Arthropathy
MRI: Magnetic resonance imaging
BMI: Body mass index
IPSG: International Prophylaxis Study Group
HJHS: Hemophilia Joint Health Scores
PFS: Progression-free survival
FVIII: Factor VIII
AJBRs: Activated partial thromboplastin time
PT: Prothrombin time
MRI-SP: MRI-detected synovial hypertrophy
PACS: Picture Archiving and Communication System
ICC: Interclass correlation coefficient
RRP: Rapid radiographic progression
